# Can Perfusion Index be used as an Objective Tool for Pain Assessment in Labor Analgesia?

**DOI:** 10.12669/pjms.345.15157

**Published:** 2018

**Authors:** Ilke Kupeli, Nur Gozde Kulhan

**Affiliations:** 1 Dr. Ilke Kupeli, Department of Anesthesiology and Reanimation, Erzincan University, Erzincan, Turkey; 2 Nur Gozde Kulhan, Department of Gynecology and Obstetrics, Erzincan University, Erzincan, Turkey

**Keywords:** Perfusion Index, Pain Assessment, Labor Analgesia

## Abstract

**Objective:**

To establish a relationship between the Visual Analog Scale for pain (VAS) in the recovery time of epidural analgesia and the Perfusion Index (PI) values at that time and to test the possibility of using PI as an objective tool for pain assessment.

**Methods:**

Thirty women were included in the study. After inserting epidural catheter, the initial applicationtime of epidural analgesia was taken as 0^th^ minute. Hemodinamics, VAS, and PIvalues were recorded at 5^th^, 10^th^, 30^th^, 60^th^ minutes and every two hours until the birth and the 30^th^ minute after the birth.

**Results:**

HR, SAP, DAP, PI, VAS values before the procedure were different than all follow-ups (p<0.001). A negative and significant correlation was found at 10^th^, 30^th^, 60^th^ minutes and 2^nd^ hour after drug administration from epidural catheter(rho:0.38; p:0.03, rho:0.47; p:0.009, rho:0.75; p<0.001, rho:0.46; p:0.009, respectively). As the pain decreased, the perfusion index increased. In 17 patients requiring additional doses, PI increased after the all medications, but a decrease was observed in the VAS values(p<0.05).

**Conclusions:**

In this study, it was determined that the pain decreased with epidural analgesia, perfusion index increased and the pain level increased significantly when the perfusion index started to decrease.

## INTRODUCTION

Labor pain is an event mechanically tiring the mother, leading stress and anxiety, and increasing oxygen demand with hyperventilation and pain is a personal and subjective experience that makes the objective measurements impossible.^1^ The length of labor, maternal pelvic anatomy, fetal size, use of oxytocin, parity, participation in birth preparation courses, fear and birth worries, pain experience and attitudes towards pain and coping mechanisms are the main factors affecting the perception of labor pain.

Many researchers have evaluated the autonomic response to pain usually by monitoring skin conductivity or heart rate.^2^,^3^ Visual Analog Scale (VAS) is the most commonly used pain assessment scale. Besides, due to the difference of psychometric stability, it has no validity in all environments.^4^

Perfusion Index (PI) is used to measure the pulse with pulse oximeter and provides an indirect and noninvasive measurement of peripheral perfusion. In the study by Nishimura et al.,^5^ on voluntary patients, they determined that the PI values examined after applying painful stimuli were correlated with the pain. This study which concluded that the increase in the sympathetic nerve tone due to pain may affect PI provides a noninvasive option for objective assessment of pain perception. This tool can eliminate psychological factors such as personality, age, gender and changes in cultural background, fear, anxiety, depression and anger for pain assessment.^6^

From this point of view, it can be suggested that sympathetic tonus increase and pain caused by the elimination of the effect of epidural analgesia applied at vaginal delivery may also affect PI. The aim of this study was to establish a relationship between the VAS value at the recovery time of the epidural analgesia and the PI values at that time and to test the possibility of using PI variations as an objective tool for predicting pain onset time and assessing pain.

## METHODS

The study was planned as an observational cohort study. The study was started after the ethics committee approval no: 03/10 of Erzincan University clinical trials ethics committee was received and informed written consents of the patients were received. ClinicalTrials.gov ID: NCT03107559.

Thirty women, aged between 18-45 years, who would have spontaneous vaginal birth were included in the study. Our exclusion criterias were not prepared to participate in the procedure, under 18 years - over 45 years of age, low platelet count, infection at the puncture site, progressive neurological disease, increased intracranial pressure, presence of hypovolaemia. The cases who had received epidural catheter and had cesarean section were excluded from the study.

After explaining the procedure to the patients by a gynecologist and the researcher anesthetist, perfusion index device with non-invasive pulse oximeter in addition to standard ASA monitorization (Heart Rate (HR), Systolic Arterial Pressure (SAP), Diastolic Arterial Pressure (DAP) were attached to all cases (Radical-7; Masimo, Tokyo, Japan). An epidural catheter was inserted after the cervical dilatation was 3-5 cm in the vaginal examination performed by the gynecologist. We preferred to insert the epidural catheter at 3-5 cm dilatation, where the VAS value of patients was five and above. An epidural catheter was inserted between L3-L4 / L4-L5 spaces after the cervical dilatation was 4-5 cm in the vaginal examination performed by the gynecologist. After making 2 cc lidocaine (Jetmonal, Adeka, Samsun, Turkey) as test dose with infusion in the epidural catheter and determining that it is the right space, 10 cc 0.25 % bupivacaine (Marcaine, AstraZeneca, UK) was applied through the epidural catheter. Before the procedure, HR, SAP, DAP, PI, and VAS values were recorded for each patient. The first application time of epidural analgesia was taken as 0th minute and these values were recorded at 5th, 10th, 30th, 60th minutes and every two hours until the birth. The hours when the patients started to feel pain and HR, SAP-DAP, VAS and PI values at these hours were recorded. All cases were followed up to the first 30 minutes after normal vaginal delivery. Epidural catheter was removed six hours after the birth.

The primary purpose of this study was to investigate the relationship between VAS values and PI in patients who underwent epidural analgesia for vaginal delivery. And the secondary purpose of the study was to investigate the relationship between PI and HR and SAP-DAP.

With this study, by examining the changes in PI, we predict that a painless birth and increased maternal satisfaction can be achieved by administering additional doses of analgesics before the patients feel any pain in the later period.

### Statistical Analysis

By taking the paper^6^ as reference, the sample size was calculated as 30 assuming α= 0.05 and the power of 85% by predicting the variation of 50% in PI. Repeated measures ANOVA was used in the comparison of means of the investigated parametersbased on time. In order to investigate the linear relationship of the data with each other, Pearson was used in the normally distributed data and Spearman correlation analysis was used in data which did not have normal distribution. In order to analyze the difference between the pre-measurements and post-measurements in the paired samples, paired t-test and its nonparametric alternative Wilcoxon signed rank test were used in dependent samples. Statistical evaluation was performed using SPSS 21 for Windows (Statistical package for the social sciences; SPSS, Chicago, IL, USA), p <0.05 was accepted as statistically significant.

## RESULTS

### Demographic Data

Thirty patients aged between 19-43 years were included in the study. The mean age of the patients was 26.2 ± 5.0. Their number of pregnancies ranged between one and 5 (1.6 ± 0.9) (Table-I). Thirteen patients gave birth within two hours after catheter insertion without the need for an additional dose. On the other hand, 17 patients needed additional doses.

**Table-I:** Demographic data.

	N	Minimum	Maximum	Mean	Std. Deviation
Age (year)	30	19	43	26.27	5.044
Number of pregnancies	30	1	5	1.63	0.964

### Hemodynamic Values

It was observed that at least one measurement in terms of HR, SAP, DAP, PI, VAS, and dilatation follow-ups was different from the other measurements (p: 0.000). According to this, HR, SAP, DAP, PI, dilatation, and VAS values before the procedure were statistically different than all follow-ups after the procedure (Table-II).

**Table-II:** The patients’ hemodynamic data, PI, servical dilatation and VAS values.

	Pre-epidural	Epidural 0. Min.	Epidural 5. min.	Epidural 10. min.	Epidural 30. min.	Epidural 60. min.	Epidural 2.hour	During delivery	Postpartum 30. min.	p
HR	106.9 ± 10.3	103.3±12.0	98.8 ± 8.6	93.1 ± 7.6	93.8 ± 7.6	96.3 ± 9.1	92.7 ± 8.9	103.3 ± 10.7	90.1 ± 5.1	0.000*
SAP	132.1 ± 14.8	131.1±12.0	123.4±11.8	119.1±13.6	120.8±14.3	117.8±15.3	124.6±13.3	130.2 ± 12.8	119.5±11.9	0.000*
DAP	82.3± 11.4	81.3 ± 9.3	77.3 ± 9.2	73.2 ± 8.8	73.7 ± 10.1	73.3 ± 10.2	75.3 ± 10.5	83.8± 12.2	70.1 ± 6.2	0.000*
PI	1.8± 1.1	2.6 ± 1.4	4.3 ± 2.2	6.9 ± 3.7	8.7 ±4 .9	9.0 ± 4.7	6.5 ± 4.2	4.5 ± 3.6	3.0 ± 1.8	0.000*
Dilatation	4.2 ± 1.0	4.3 ± 1.0	4.5 ± 1.1	5.0 ± 1.6	6.0 ± 1.7	6.4 ± 2.0	7.8 ± 2.1	10.0 ± 0.0		0.000*
VAS	7.7 ± 1.3	7.4 ± 1.3	5.5 ± 1.3	3.6 ± 1.6	2.5 ± 1.6	2.7 ± 1.9	4.6 ± 1.8	5.3 ± 1.1	1.2 ± 0.4	0.000*

* Repeated measures ANOVA. The mean difference is significant at the 0.05 level.

(HR: heart rate, SAP: systolic arterial pressure; DAP: diastolic arterial pressure; PI: perfusion index; VAS: visual analogue scale)

### Perfusion Index and VAS Data

**Graph.1:**
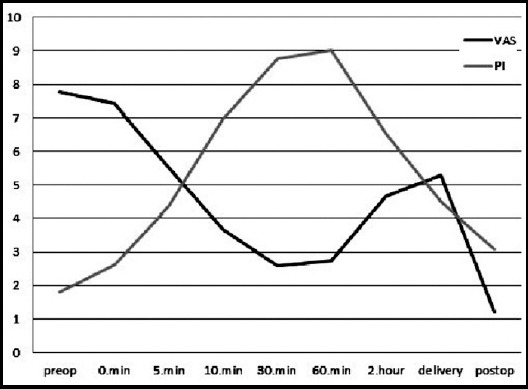
Relationship between PI and VAS. Graph.1 There was neagative but statistacally significant correlation bewteen the VAS and PI at the 10^th^, 30^th^, 60^th^ minutes and 2^nd^ hour after the drug administration from epidural catheter (rho:0.38; p:0.03, rho:0.47; p:0.009, rho:0.75; p:<0.001, rho:0.46; p:0.009, respectively)

In the data of the relationship between VAS and PI which is the main objective of our study; when all patients were taken into consideration;there was a negative but insignificant correlation between the VAS and PI before the procedure and at the 0th and 5th minutes (rho:0.21, p:0.26). However, there was a negative but statistically significant correlation at the 10th, 30th, 60th minutes and 2nd hour after the drug administration from epidural catheter (rho:0.38, p:0.03; rho:0.47, p:0.009; rho:0.75, p<0.001; rho:0.46, p:0.009; respectively) (Graph.1).

Similar condition was observed at the 10^th^, 30^th^ minutes, and at 2^nd^ hour. However, a negative but insignificant correlation was found at 4th hour, 8th hour, in the birth and in the postnatal follow-ups (p > 0.05). Seventeen out of 30 patients were found to require additional doses. When the values before and after the additional dose conducted were compared, it was observed that there was a significant difference between the VAS values before and after the drug administration (p < 0.001). There was also a significant difference in PI values before and after the drug administration (p < 0.001) (Table-III).

**Table-III:** Visual analogue scale (VAS) and PI values before and after the drug administration.

	N	Mean	Std. Deviation	p
pre-drug VAS	17	6.65	1.5	<0.001*
post-drug VAS	17	3.35	1.6	
pre-drug PI	17	3.35	3.1	<0.001*
post-drug PI	17	6.59	4.1	

*Wilcoxon, The mean difference is significant at the 0.05 level.

### The Relationship Between Perfusion Index and Other Parameters

When examining the correlation of perfusion index with HR, and SAP-DAP; a negative but significant correlation was determined between PI and HR before the procedure and at the 0th and 5th minutes (rho: 0.49, p: 0.005; rho: 0.42, p: 0.021; rho: 0.58, p: 0.001, respectively). However, this correlation was not significant in the subsequent follow-ups. Perfusion index had no significant correlation with SAP and DAP (p > 0.05).

## DISCUSSION

**Graph.2:**
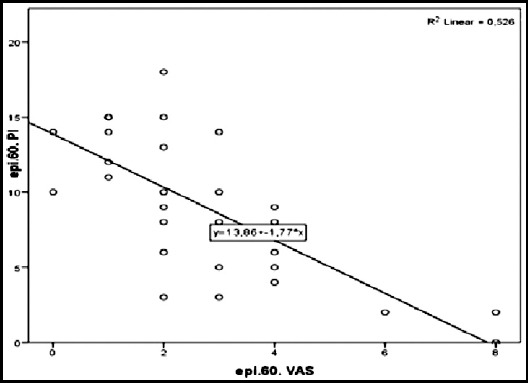
Relationship between PI and VAS 2.

In this study investigating the relationship between pain level and perfusion index in labor analgesia for vaginal delivery, it was determined that as the pain decreased with epidural analgesia, perfusion index increased and when the perfusion index started to decrease, the pain level increased significantly (Graph.2).

Birth has become one of the most painful experiences known, and physical, emotional, psychological, sociological, and sometimes religious beliefs should be taken into account along with when considering treatment. Epidural analgesia is a highly effective and popular treatment for labor pain.^7^ Epidural analgesia provides more analgesia in the parenteral opioid in the first and second stages of labor and results in less neonatal depression.^7^,^8^ Since sympathetic fibers are those that are affected the most physiologically in epidural analgesia, we can describe the epidural block as a kind of ’chemical sympathectomy’.^9^ This results in a reduced sympathetic response to the painful stimulus such as reduced heart rate and blood pressure response. In the present study, a decrease was also observed in heart rate, SAP, and DAP after the drug administration through epidural catheter.

Pain affects the endocrine system leading to increased catecholamine release resulting in vasoconstriction. A number of studies have shown that the application of painful stimuli induces the sympathetic nervous system resulting in increased heart rate,^10^ skin conduction^11^ and vasomotor tone.^12^,^13^ In epidural analgesia, vasodilatation is seen in the lower part of the body when sympathetic stimulus disappears. As a result of this vasodilatation, blood flow to the lower part of the body increases and venous pooling occurs. Perfusion index reflects the ratio of pulsatile blood flow to non-pulsatile blood flow at the monitoring site. Perfusion index is a continuous measure of indirect, noninvasive, and peripheral perfusion. Changes in sympathetic nerve tone may affect smooth muscle tone and change perfusion level.^14^ Temperature, volume, and anesthesia can affect perfusion in the extremities by causing vasoconstriction and vasodilatation, which can lead to a decrease in PI or an increase in PI, respectively.^6^ In the present study, PI also increased significantly due to vasodilatation in extremities after the epidural in parallel with the literature.

In the literature, it is possible to find some studies in which autonomic responses are evaluated by pulse oximetry.^15^,^16^ In these studies, it was mentioned that changes can be observed with painful stimuli in PI even in general anesthesia. Similarly, in the study conducted by Nishimura et al.^5^ with healthy people to evaluate the advantages and limits of PI as a complementary pain assessment tool for developing better pain evaluation guidelines, they observed that PI decreased with painful electrical stimulation. In the study conducted by Mohamed et al.^6^ to investigate the postoperative PI and VAS relation, it was determined that as VAS values increased, PI decreased but there was a significantly increased PI after the analgesia. Similar to the literature, in the present study, it was observed that PI was low when the VAS values were high and an increase was observed in PI along with the decrease in VAS values after the epidural analgesia. A negative correlation was determined between VAS and PI. This suggested that both epidural analgesia was successful; therefore, the patient’s pain was relieved and also there was an increase in perfusion, and thus in PI, with the increase in blood flow to the lower extremity due to epidural sympathetic blockade.

When other sympathetic system results that may affect the perfusion index were examined, a few studies are seen in the literature but they have contradictory results. Nishimura et al.,^5^ determined in their study that the changes in PI were independent from heart rate. Mohamed et al.,^6^ suggested that there was a decrease in the VAS, HR and the mean arterial pressure and an increase in PI as a result of the adequate relief in the pain. In the present study, there was a negative but significant correlation between HR and PI before the analgesia was provided. As the heart rate increased, the perfusion index decreased. However, this correlation was not significant in subsequent follow-ups after the analgesia was provided. Again, the perfusion index had no significant correlation with SAP and DAP in this study. This suggested that PI was more affected by the stressful stimuli and pain rather than the changes in heart rate.

### Limitations of the study

The first one is that the sample size was small. Generalization cannot be made since follow-ups were performed only in 17 patients out of 30 patients. It is thought that more reliable results can be obtained with higher number of patients. The second one is that we attribute the lack of the correlation between VAS and PI after the 4^th^ hour to the number of patients. This is because 17 patients reached 4^th^ and 8^th^ hours. There is a need for studies in which all patients are followed up for the same period. The third limitation is that the anxiety level of the patients was not considered. However, fear and birth worries, pain experience, attitudes towards pain and coping mechanisms are the main factors affecting the perception of labor pain.

## CONCLUSION

In this study investigating the relationship between pain level and perfusion index in labor analgesia for vaginal delivery, it was determined that when the pain decreased with epidural analgesia, the perfusion index increased, and when the perfusion index started to decrease, the pain level increased significantly. According to the results of this study, it is thought that PI may be an independent parameter reflecting the perception of painful stimuli and may offer a noninvasive option to objectively assess pain perception.
